# Supplementary material for: Simply saliva: stability of SARS-CoV-2 detection negates the need for expensive collection devices
Source: medRxiv. 2020 Aug 4:2020.08.03.20165233. Preprint. [Version 1] doi: 10.1101/2020.08.03.20165233 (PMC7418742; doi:10.1101/2020.08.03.20165233)
Supplement: 1 [file NIHPP2020.08.03.20165233-supplement-1.pdf]

medRxiv preprint doi: https://doi.org/10.1101/2020.08.03.20165233.this version posted August 4, 2020. The copyright holder for this preprint (which was not certified by peer review) is the author/funder, who has granted medRxiv a license to display the preprint in perpetuity.
It is made available under a CC-BY 4.0 International license .

Supplemental Figures

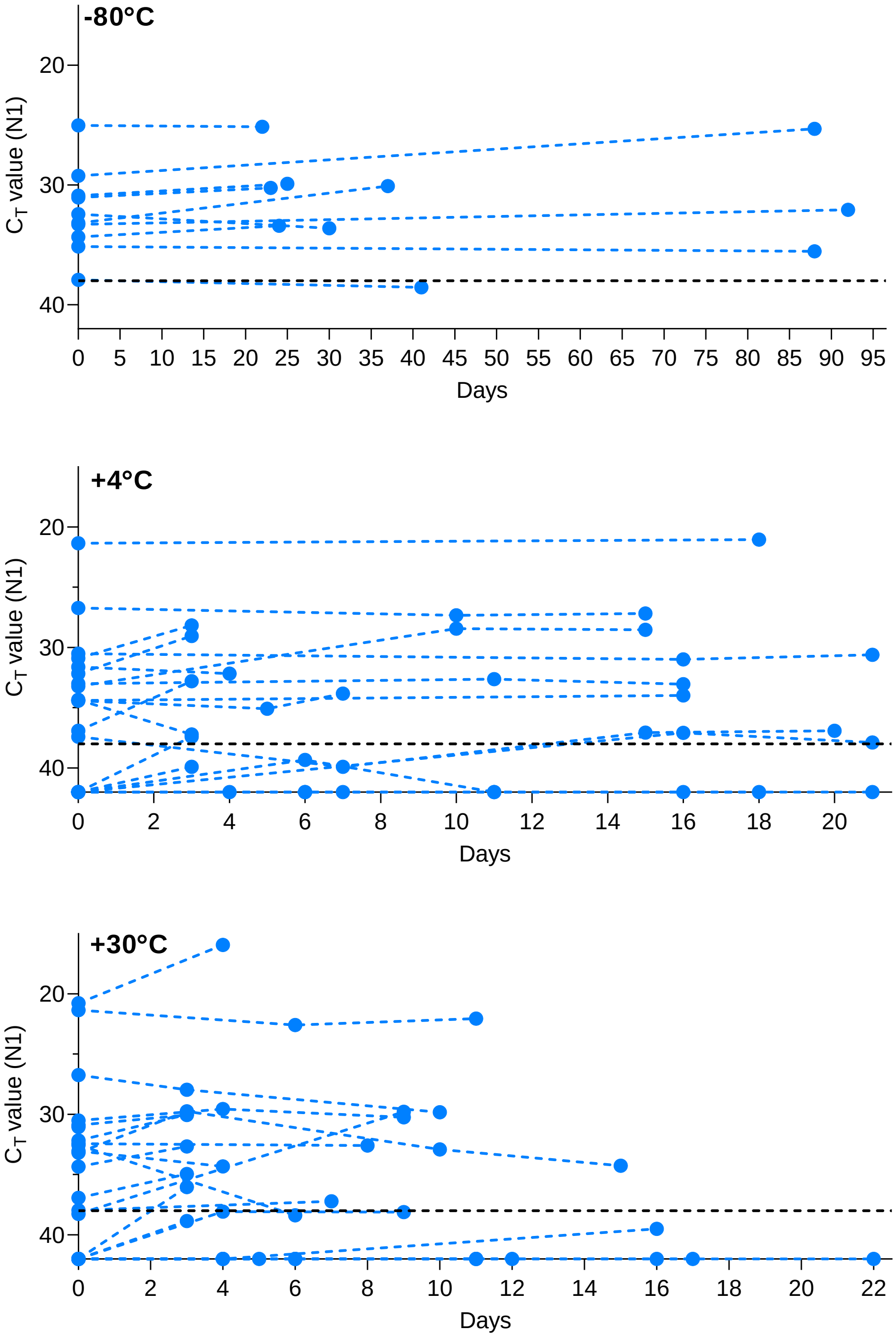

Supplemental Figure 1. **Stability of SARS-CoV-2 RNA detection in saliva.** SARS-CoV-2 RNA detection in saliva on day of sample collection (0) or after prolonged storage at -80°C, 4°C or 30°C. Ct values from the same original sample are connected by a dotted line. The -80°C and 4°C conditions were found to have a weakly beneficial effect on signal detection by the mixed effects model, while the 30°C condition resulted in a slight increase in Ct. The -80°C storage alone did not cross zero suggesting a mildly stronger effect than the other conditions (95% CI: -0.038, -0.010). The black dashed line represents Ct 38 which we applied as the cut-off to determine sample positivity. Samples that remained not detected (ND) after 45 cycles are depicted as Ct 42.

medRxiv preprint doi: https://doi.org/10.1101/2020.08.03.20165233.this version posted August 4, 2020. The copyright holder for this preprint (which was not certified by peer review) is the author/funder, who has granted medRxiv a license to display the preprint in perpetuity.
It is made available under a CC-BY 4.0 International license .

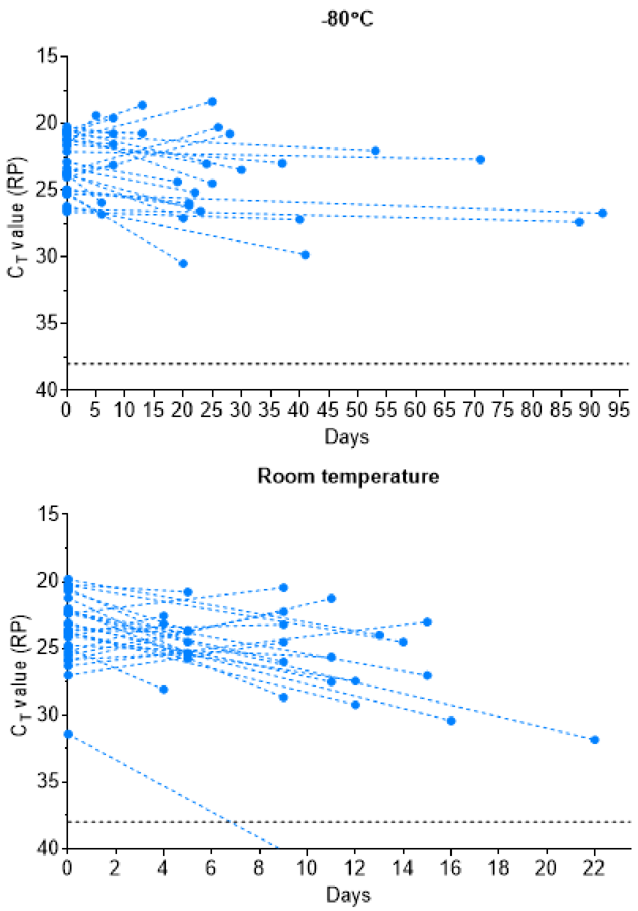

Supplemental Figure 2. **Detection of human *RNAse P* (RP) declines over time when stored in saliva in warmer conditions.** Detection of human RP in saliva on day of collection (0) or after prolonged storage at -80°C, 4°C, room temperature (~19°C) or 30°C. Ct values from the same original sample are connected by a dotted line. Prolonged storage at -80°C and 4°C had minimal effect on RP detection with Ct changes of 0.832 (95% CI: -0.402, 2.038) and -0.315 (95% CI: -2.336, 1.687), respectively. However, storage at room temperature (Ct +1.837, 95% CI: 0.468, 3.188) and 30°C (Ct +3.526, 95% CI: 1.750, 5.349) was detrimental to RP, exhibiting a more substantial decrease in signal at these warmer conditions. The black dashed line represents Ct 38 which we applied as the cut-off to determine sample positivity. Samples that remained not detected (ND) after 45 cycles are below the y-axis limit.

medRxiv preprint doi: https://doi.org/10.1101/2020.08.03.20165233.this version posted August 4, 2020. The copyright holder for this preprint (which was not certified by peer review) is the author/funder, who has granted medRxiv a license to display the preprint in perpetuity.
It is made available under a CC-BY 4.0 International license .

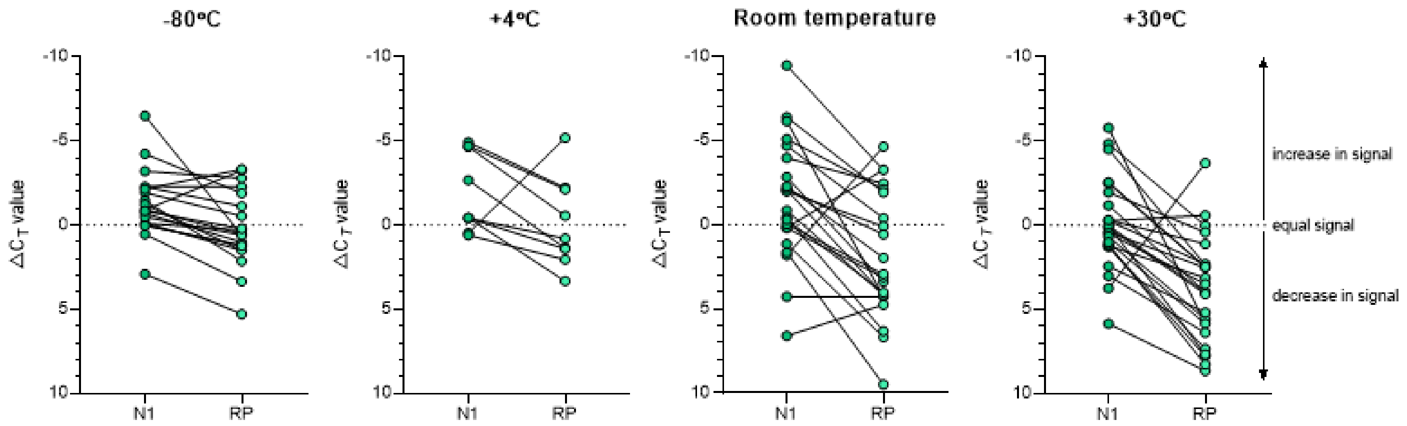

Supplemental Figure 3. Detection of SARS-CoV-2 RNA (N1) in saliva remained more stable over time than human *RNAse P* (RP). Delta Ct was calculated as the difference in Ct value from the day of saliva collection and after storage at -80°C, 4°C, room temperature (~19°C) or 30°C. Delta Ct values from the same sample are joined by a solid line. While the change in detection of SARS-CoV-2 N1 and RP was similar in saliva samples stored at 4°C (Wilcoxon signed rank test, p = 0.129), a greater difference was observed between the change in N1 and RP for samples stored at -80°C (p = 0.001), room temperature (p = 0.001) and 30°C (p < 0.0001).

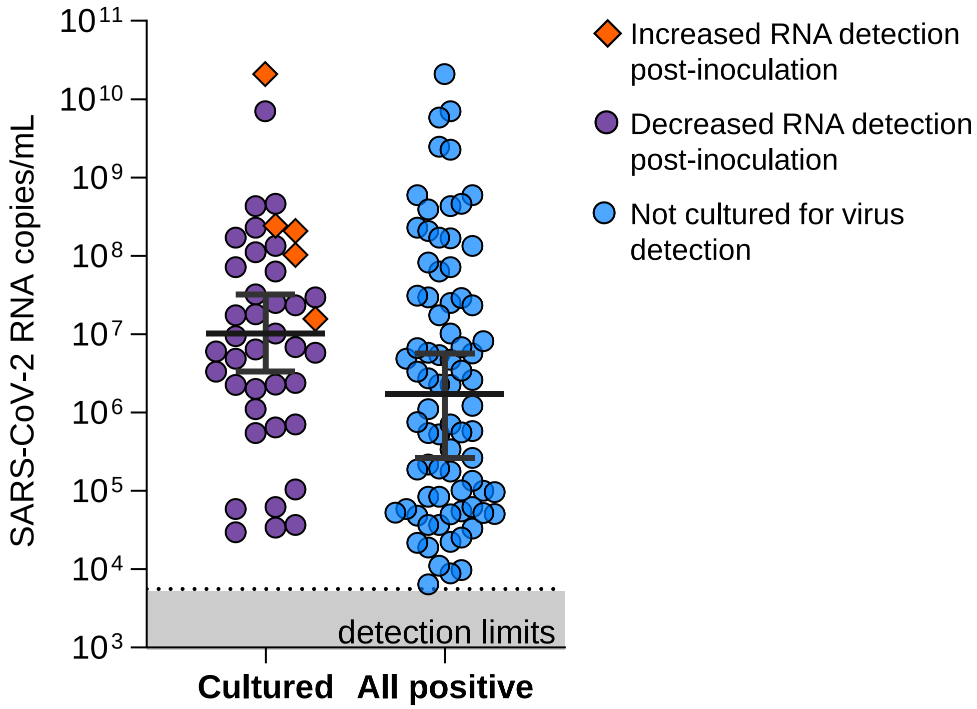

Supplemental Figure 4. Saliva samples of relatively high viral load were cultured to evaluate the infectiousness of SARS-CoV-2 in saliva. Saliva samples cultured on Vero-E6 to test for infectious virus were of higher SARS-CoV-2 RNA (N1) load as compared to the overall saliva samples collected by Yale IMPACT[2] which tested positive for SARS-CoV-2 (Mann-Whitney, p = 0.0136). Orange diamonds denote samples in which we observed an increase in viral RNA detection 72 hours post-inoculation.
